# Is there any association between vitamin D levels and polycystic ovary syndrome (PCOS) phenotypes?

**DOI:** 10.20945/2359-3997000000177

**Published:** 2019-09-25

**Authors:** Maryam Eftekhar, Elham Sadat Mirhashemi, Behnaz Molaei, Soheila Pourmasumi

**Affiliations:** 1 Research and Clinical Center for Infertility Yazd Reproductive Sciences Institute Shahid Sadoughi University of Medical Sciences Yazd Iran Research and Clinical Center for Infertility, Yazd Reproductive Sciences Institute, Shahid Sadoughi University of Medical Sciences, Yazd, Iran; 2 Abortion Research Center Yazd Reproductive Sciences Institute Shahid Sadoughi University of Medical Sciences Yazd Iran Abortion Research Center, Yazd Reproductive Sciences Institute, Shahid Sadoughi University of Medical Sciences, Yazd, Iran; 3 Department of Obstetrics and Gynecology, Fellowship of Perinatology Zanjan University of Medical Sciences Zanjan Iran Department of Obstetrics and Gynecology, Fellowship of Perinatology, Zanjan University of Medical Sciences, Zanjan, Iran; 4 Non-Communicable Diseases Research Center Rafsanjan University of Medical Sciences Rafsanjan Iran Non-Communicable Diseases Research Center, Rafsanjan University of Medical Sciences, Rafsanjan, Iran; 5 Clinical Research Development Unit (CRDU) Moradi Hospital Rafsanjan University of Medical Sciences Rafsanjan Iran Clinical Research Development Unit (CRDU), Moradi Hospital, Rafsanjan University of Medical Sciences, Rafsanjan, Iran

**Keywords:** Polycystic ovary syndrome (PCOS, Rotterdam criteria, vitamin D

## Abstract

**Objective:**

The aim of this study was to assess the serum vitamin D level in a retrospective study in women with polycystic ovary syndrome (PCOS), according to the different phenotypes of the disease.

**Subjects and methods:**

In this retrospective study, the records of 351 infertile women who were diagnosed with PCOS were examined, and 200 of them were enrolled in the study randomly in 4 PCOS phenotypes. Fifty normal ovulatory women with the history of male factor were selected as the control group. Parameters, including age, infertility duration, body mass index (BMI), hormone profile, as well as the serum vitamin D level were compared among the 4 phenotypes, with the P-value ≤ 0.05 considered statistically significant.

**Results:**

The findings showed a higher serum vitamin D level in the control group than in PCOS patients, which was statistically significant (P < 0.001). In addition, there was no significant difference in the serum vitamin D level among the four phenotypes of PCOS.

**Conclusions:**

No significant difference was found in the serum vitamin D level of the different phenotypes of PCOS. Further studies with larger sample sizes are recommended to be done to establish the role of the serum vitamin D level in PCOS patients.

## INTRODUCTION

Polycystic ovary syndrome (PCOS) is referred to the conditions that affect the endocrine system. About 5-21 percent of women at the reproductive age are at the risk of this fertility problem. The major common symptoms of PCOS are a defect in the menstrual cycle, ovulatory deficiencies, and hirsutism. PCOS is a disorder of various types, with metabolic syndromes, including hyperandrogenemia, dyslipidemia, and insulin resistance being the other characteristics of this disease (1).

The Rotterdam criteria are the guidelines for the diagnosis of PCOS. These criteria are highly accepted for the diagnosis of PCOS, with this disease being diagnosed typically in women who show at least two of the three symptoms of I; oligo or anovulation, II; hyperandrogenemia, and III; poly cysts in the ovaries on ultrasonography (2).

PCOS can increase the infertility risk and affect the patient’s health in many ways (3). The term ‘polycystic ovarian syndrome’ does not reﬂect the complications of this disorder fully or correctly. It involves a wide range of clinical expressions and related diseases. Women with PCOS have the indications of reproductive defects, including insulin resistance; in addition, they are at a higher risk of type 2 diabetes mellitus, high blood pressure, low HDL, high LDL, depression, and anxiety (4).

Pregnant women with PCOS are at a high risk of gestational diabetes, preeclampsia, fetal macrosomia, small-for-gestational age infants, and perinatal mortalities (5).

In the past decades, signiﬁcant attempts were made to categorize PCOS types. The initial data included chronic anovulation and hyperandrogenism as the indicators of PCOS classification. In the end, the collected data were integrated into the Rotterdam criteria (6). Based on the Rotterdam criteria, four different phenotypes can be defined for PCOS, including hyperandrogenism, chronic anovulation, and polycystic ovaries (PCO); hyperandrogenism and chronic anovulation but normal ovaries; hyperandrogenism and polycystic ovaries but ovulatory cycles; and chronic anovulation and polycystic ovaries but no clinical or biochemical hyperandrogenism (7).

Clinical researchers have recently focused on vitamin D supplementation as an adjuvant treatment for PCOS. It has been confirmed that women with PCOS have a high level of vitamin D deﬁciency. Hence, a correlation has been established between the serum vitamin D level and several metabolic symptoms in PCOS patients. It has recently been suggested that vitamin D deficiency may be responsible for the pathogenesis of PCOS (8). Numerous studies have shown that the biomarker of vitamin-D, i.e. 25-hydroxy vitamin D (25 OH D), is negatively correlated with the body fat level, waist circumference, and the body mass index (BMI) (9-11). In the literature review for the present study, we found a paper that examined the serum vitamin D level in various phenotypes of PCOS. Davis and cols. in this paper evaluated vitamin D level in Intrauterine insemination (IUI) cases based on PCOS phenotype (12).

In the present retrospective study, we assessed the serum vitamin D level in women with PCOS, according to the different phenotypes of the disease.

## SUBJECTS AND METHODS

In this retrospective cohort study, over a 30-month period from April 2015 to August 2017, the clinical and laboratory records of 3000 patients were reviewed. The present study was approved by the Ethics Committee of Yazd Reproductive Sciences Institute, Shahid Sadoughi University of Medical Sciences, Yazd, Iran, under the ethics code of IR.SSU.RSI.REC.1396.31. Three hundred and fifty one infertile women aged 18-40 were diagnosed with PCOS. Next, 50 cases in each phenotype group (200/351 women) were enrolled randomly in the study. Fifty normal ovulatory women with the history of male factor were then selected as the control group. In control group women with regulatory menses, normal ovarian reserve (AMH>1.2 ng/mL) and non PCOS based on Rotterdam criteria selected.

Inclusion criteria: women aged between 18-40 years old were diagnosed with polycystic ovary syndrome based on Rotterdam criteria,

Exclusion criteria: women with history of endometriosis, hyperprolactinemia, recurrent abortion, and sever male factor including testicular sperm extraction (TESE), percutaneous epididymal sperm aspiration (PESA), oligospermia were excluded.

PCOS was diagnosed based on the Rotterdam criteria (2). PCOS would be diagnosed if at least two of the following criteria were met: ‘oligomenorrhea/anovulation’ (defined as the delay in menses over 35 days or less than 8 spontaneous hemorrhagic episodes per year), clinical and/or biochemical hyperandrogenism (defined as the total circulating testosterone levels being over 95 percent (0.481 ng/mL) of the levels detected in the group of women with no clinical evidence of hyperandrogenism or menstrual disturbances and taking no hormonal medication), and polycystic ovary on ultrasonography (≥ 12 small follicles measuring 2-9 mm in at least one ovary and/or the ovarian volume > 10 cm^3^).

The patients were categorized into four groups, according to the following defined criteria: Hyperandrogenism, chronic anovulation and polycystic ovaries; hyperandrogenism and chronic anovulation but normal ovaries; hyperandrogenism and polycysticovaries but ovulatory cycles; and chronic anovulation and polycystic ovaries but no clinical or biochemical hyperandrogenism. The data were collected from the hospital records. All hormonal and vitamin D level were tested in the hematology laboratory of Yazd research and clinical center for infertility. Biochemical assay to determine vitamin D level was VD3 (Vitamin D3) ELISA Kit (Elabscience; E-EL-0014) and detection range of this test was between 1.56 to 100 ng/ml. Other hormones were determined by specific ELISA kits.

The patients’ data included age, duration of infertility, body mass index (BMI), hormone profile, and the serum vitamin D level.

### Statistical analysis

Data was analyzed using Statistical Package for the Social Sciences 20.0 (SPSS, SPSS Inc, Chicago, Illinois). Continuous data were presented as mean ± standard deviation (SD) and assessed by independent Student’s t-test. Analysis of variance (ANOVA) with post hoc LSD were used to comparison between mean of parameters pair ways. P-value less than 0.05 considered statistically significant.

## RESULTS

The data from 200 PCOS cases were analyzed in this study and compared to 50 male factor infertility cases as the control group. The patients’ characteristics are summarized in Table 1. The mean age and duration of infertility were similar in the two groups. The statistical analysis demonstrated a higher serum vitamin D level in the control group than in PCOS patients, having been statistically significant (P < 0.001). Other factors, including body mass index (BMI), anti-mullerian hormone (AMH), follicle stimulating hormone (FSH), luteinizing hormone (LH), and fasting blood sugar (FBS) were significantly different between the two groups (Table 1).

**Table 1:** Comparison of Women’ characteristics between PCOS patients and control

p-value	Control (n = 50)	PCOS (n = 200)	Variables
NS	28.46 ± 4.18	28.19 ± 5.37	Women’s age (years)
NS	5.44 ± 3.53	5.82 ± 3.78	Infertility duration (years)
0.001	29.08 ± 5.50	20.60 ± 9.22	Vit D3 (ng/mL)
0.001	3.76 ± 1.22	8.70 ± 4.07	AMH (ng/mL)
0.001	23.96 ± 2.63	28.54 ± 3.45	BMI (kg/m^2^)
0.001	5.36 ± 2.96	9.14 ± 5.51	LH (IU/l)
0.001	6.66 ± 1.69	5.42 ± 2.21	FSH (IU/l)
0.001	0.88 ± 0.44	1.97 ± 2.06	LH/FSH ratio ≥2.5
0.001	97.56±14.12	99.16±17.76	FBS (mg/dl)

Note: Values are presented by mean ± SD. P-values obtained from difference between means were tested for significance by one way ANOVA on ranks.

PCOS: polycystic ovarian syndrome; AMH: anti-mullerian hormone; BMI: body mass index; LH: luteizing Hormone; FSH: follicle stimulating hormone; FBS: fasting blood sugar.

As Table 2 shows, the parameters were compared among PCOS phenotypes. There was no significant difference in the serum vitamin D level among the four phenotypes of PCOS. Three parameters of AMH, LH, and testosterone were significantly different among the four phenotypes. Significant differences of the parameters and the results of the comparison between the PCOS phenotypes pair ways showed by letters. Significant differences were observed between A and B phenotypes (P-Value = 0.02) in terms of AMH. In addition, LH was significantly different between A and C phenotypes (P-Value = 0.007), and also between A and D phenotypes (P-Value = 0.08). There was also a significant difference between B and C phenotypes in terms of the FBS parameter (P-Value = 0.008) (Table 2). The analysis results of comparison between vitamin D levels in associate with PCOS variables showed that there was no significant difference between the serum vitamin D level and the study parameters (Table 3).

**Table 2:** Comparison of study variables in four PCOS phenotypes

p-value	Phenotype D (n = 50)	Phenotype C (n = 50)	Phenotype B (n = 50)	Phenotype A (n = 50)	Variables
NS	27.20 ± 6.13	28.12 ± 4.81	29.44 ± 4.57	27.98 ± 5.71	Women’s age (years)
NS	5.28 ± 4.04	6.00 ± 3.91	6.08 ± 3.40	5.90 ± 3.80	Infertility duration (years)
NS	19.97 ± 9.33	20.54 ± 9.53	20.92 ± 9.43	20.97 ± 8.80	Vit D3 (ng/mL)
0.001	9.71 ± 5.39^b^	8.53 ± 3.33	7.13 ± 2.35^a, b^	9.43 ± 4.19^a^	AMH (ng/ml)
NS	28.27 ± 4.30	28.44 ± 3.57	28.43 ± 3.03	29.00 ± 2.75	BMI (kg/m^2^)
0.01	8.54 ± 5.46^d^	7.61 ± 5.07^c^	9.26 ± 4.87	11.14 ± 6.12^c, d^	LH (IU/l)
NS	5.39 ± 2.26	5.19 ± 1.88^e^	5.94 ± 2.62^e^	5.16 ± 1.97	FSH (IU/l)
NS	2.16 ± 3.48	1.52 ± 1.06	1.73 ± 0.92	2.41 ± 1.66	LH/FSH ratio
0.01	98.04 ± 10.66	102.30 ± 21.85	91.86 ± 12.85	98.52 ± 16.73	FBS (mg/dl)
0.001	0.56 ± 0.28^f^	0.75 ± 0.41	0.94 ± 0.35	0.91 ± 0.38^f^	Testosterone (IU/l)

Note: values are presented by mean ± SD. P-values obtained from difference between means were tested for significance by one way ANOVA on ranks. Different letters indicate statistical difference for comparisons between PCOS phenotypes. ^a^: Significant between A and B phenotypes. ^b^: Significant between B and D phenotypes. ^c^: Significant between A and C phenotypes. ^d^: Significant between A and D phenotypes. ^e^: Significant between B and C phenotypes. ^f^: Significant between A and D phenotypes.

AMH: anti-mullerian hormone; BMI: body mass index; LH: luteizing hormone; FSH: follicle stimulating hormone; FBS: fasting blood sugar.

**Table 3:** Comparison between vitamin D levels in associate with PCOS variables

p-value	Toxicity level (>100 ng/mL) (n = 0)	Sufficiency (30 to 100 ng/mL) (n = 74)	Insufficient (20 to <30 ng/mL) (n = 99)	Deficiency (<20 ng/mL) (n = 27)	Variables
NS	-	28.04 ± 5.10	28.30 ± 5.91	28.15 ± 3.93	Women’s age (years)
NS	-	5.36 ± 3.58	6.30 ± 5.82	5.26 ± 4.07	Infertility duration (years)
NS	-	8.61 ± 4.08	8.73 ± 3.80	8.84 ± 5.09	AMH (ng/mL)
NS	-	28.22 ± 2.86	28.76 ± 3.21	28.57 ± 5.35	BMI (kg/m^2^)
NS	-	8.74 ± 5.57	9.06 ± 5.41	10.51 ± 5.73	LH (IU/l)
NS	-	5.29 ± 2.09	5.44 ± 2.35	5.68 ± 2.22	FSH (IU/l)
NS	-	1.80 ± 1.14	2.09 ± 2.70	2.02 ± 1.20	LH/FSH ratio
NS	-	98.68 ± 12.91	97.05 ± 17.58	97.26 ± 20.59	FBS (mg/dL)
NS	-	0.85 ± 0.39	0.77 ± 0.36	0.71 ± 0.44	Testosterone (IU/l)

Note: values are presented by mean ± SD. P-values obtained from difference between means were tested for significance by one way ANOVA on ranks.

AMH: anti-mullerian hormone; BMI: body mass index; LH: luteizing hormone; FSH: follicle stimulating hormone; FBS: fasting blood sugar.

## DISCUSSION

This study focused on the serum vitamin D level and the prevalence of vitamin D deficiency in patients with different phenotype of PCOS. The results showed a significant difference in the total serum vitamin D level and the occurrence of vitamin D deficiency between women with PCOS and the controls. However, there was no significant difference in the serum vitamin D level among various PCOS phenotypes.

Since a great deal of evidence verifies that vitamin D plays an essential role in reproductive activities, all patients in the present study were infertile (13). Studies have verified the presence of the receptors of vitamin D in many tissues and in the reproductive system, including ovaries, the endometrium, and the placenta (14). It has been proved that the vitamin D deficiency is related to calcium dysregulation. This condition is responsible for the increase in the follicular arrest and results in menstrual and fertility disorders in women with PCOS (12).

Consistent with the findings of the present study, Wehr and cols. (15) reported that the serum vitamin D level in women with PCOS (n = 545) was lower than that of the control group (n = 145), being 25.7 and 32 ng/mL, respectively. A substantial number of studies suggest that serum vitamin D levels are similar in women with and without PCOS (16-18). In the search for the correlation between vitamin D and PCOS, only one study was found that reported women with PCOS had a significantly higher serum vitamin D level than the control women, with the similar age and BMI (19). Thus, there are varied findings in the literature about serum vitamin D levels in women with and without PCOS.

Newly Davis and cols. in 2018 published a paper and evaluated vitamin D level in all PCOS cases and male infertility as control group in intrauterine insemination (IUI) cycles. They reported in androgen excess patients level of vitamin D was lower than other PCOS phenotypes (12). This finding was in contrast to our result however they don’t show the androgen level to find androgen excess but we presented testosterone level as androgen in 4 phenotypes. In our results androgen level in patients who had vitamin D deficiency was lower than other categories of vitamin D however this difference was not statistically significant (Table 3). Also we presented fertility hormones level in 4 phenotypes and this was the strengths of the study.

Kim and cols. reported that in PCOS patients with vitamin D deficiency, a 2-month treatment with 1500 mg calcium on a daily basis and 50,000 units of vitamin D on a weekly basis improved menstrual cycles in 7 out of 9 cases (18). In the same vein, some other studies reported that the serum vitamin D level was negatively correlated with BMI, body fat, insulin resistance, and hyperinsulinemia; they also reported a negative correlation between the serum vitamin D level and metabolic disorders in PCOS patients (20-22).

Research has found a correlation between PCOS and other metabolic problems, including high blood pressure, depression and anxiety, dyslipidemia, and chronic inflammation. It has also been verified that metabolic disorders are common in women with PCOS (23). Hang Wun and cols., in their study, stated that vitamin D deficiency was highly prevalent in women with PCOS in Scotland, with this rate having been higher than that of ovulatory control women and the general UK population. They also demonstrated that vitamin D deficiency in PCOS women was correlated with metabolic risk factors, including insulin resistance and low HDL-C levels, independent of obesity measures (17).

Although we did not find any study evaluating the serum vitamin D level in different phenotypes of PCOS in the literature review of the present study, there were some studies that evaluated the correlation between PCOS and clinical manifestations. In their study, Reza Ghadimi and cols. concluded that although hypovitaminosis D was common in PCOS patients, it did not correlate with the clinical features or complications of obesity and insulin resistance. However, it was established that in PCOS patients, there was a correlation between the severity of vitamin-D deficiency and some features and complications of PCOS, including obesity and insulin resistance (24).

Vakili and cols. conducted a study on 260 PCOS women (cases) and 221 normo-ovulatory women (controls), who were recruited from a reproductive endocrinology clinic. They classified the cases into the two groups of severe and mild PCOS phenotypes, based on their clinical and para-clinical features. The adenosine to guanine single nucleotide polymorphismn of the VDR gene (rs757343) was genotyped using the PCR–RFLP method. They reported that the distribution of genotypes and alleles did not change between the cases and the controls, indicating that the single nucleotide polymorphism (SNP) was not correlated with the increased risk of PCOS. However, the SNP was responsible for the severity of PCOS phenotypes. In cases that an allele was present, the risk of a severe phenotype was by 74% higher than in other patients (OR, 1.74; 95% CI, 1.07–2.82). They also showed that the genetic variant of the VDR was correlated with the severity of the clinical appearance of PCOS, but not with the chance of PCOS occurrence (25). In a genetic study by Dasgupta and cols. they evaluated the correlation between VDR and PCOS in Indian patients. They found a significant correlation between the genotypes of VDR and some PCOS manifestations (26).

In conclusion, we did not find any significant differences in vitamin D level among different phenotypes of PCOS. Further studies with larger samples size are recommended to conclusively establish the role of serum vitamin D level on PCOS patients, particularly including data PCOS phenotypes. This study was retrospective and this was the first limitation of study and Insulin resistance and metabolic abnormalities have been associated with vit D deficiency in PCOS and the lack of these data in the manuscript was the limitation of the study.
